# Prostate Cancer – To screen, or not to screen, is that the question?

**DOI:** 10.1186/1471-2490-8-20

**Published:** 2008-12-23

**Authors:** Charles J Rosser

**Affiliations:** 1 Department of Urology and Pharmacology and Therapeutics, University of Florida, Gainesville, Florida, USA

## Abstract

There continues to be controversy regarding serum Prostate-Specific Antigen (PSA) and prostate cancer screening. We anxiously await the results of two large prospective randomized clinical trials (Prostate, Lung, Colon, and Ovary-PCLO screening trial in the US and European Randomized Study of Screening for Prostate Cancer-ERSPC in Europe) assessing the benefits of prostate cancer screening. However the true question to answer may be which cancer to treat and when should we treat it.

## 

After the introduction of serum PSA over 20 years ago as a tumor marker for prostate cancer, controversy regarding PSA and prostate cancer screening still abounds. First, who should be screened? Second, does screening affect mortality? Third, serum PSA used for screening has a low specificity (~30%) thus a vast number of patients are undergoing costly and invasive procedures to diagnosis prostate cancer patients. Fourth, does screening lead to over diagnosis and over treatment? These are just a few of the controversial issues surrounding PSA and prostate cancer screening.

It is clear that over the past decade, the utility of serum PSA in diagnosing prostate cancer has declined [1]. Though still an important screening tool for prostate cancer, we have noticed a 'PSA migration' (i.e., heavily screened populations are presenting with lower serum PSA levels today compared to 10–20 years ago) [2]. Though serum PSA may not be the ideal screening tool, it is the centerpiece of two large prospective randomized clinical trials (Prostate, Lung, Colon, and Ovary-PCLO screening trial in the US and European Randomized Study of Screening for Prostate Cancer-ERSPC in Europe) assessing the benefits of prostate cancer screening [3]. Though we are lacking level I evidence demonstrating the benefit of screening, we are engulfed in a sea of circumstantial evidence associated with prostate cancer screening. First in two large European studies, prostate cancer survival improved in men who underwent prostate cancer screening and treatment compared to those who did not undergo screening and/or treatment [4,5]. Similarly, in the Olmstead County (Minnesota) study, routine prostate cancer screening was associated with lower mortality rates than in years prior to serum PSA testing [6]. Prostate cancer has held the dubious distinction for two decades of being the second leading cause of cancer related deaths in American men over the age of 45 years. However, since the advent of PSA screening in the late 1980s, mortality rates of prostate cancer have steadily declined for the past decade. In 2008, it is estimated that 28,660 men will succumb to prostate cancer. This number is reminiscent of prostate cancer mortality rates from the 1940–1970's [7].

Though routine prostate cancer is controversial, the controversy is decreased when we consider screening in African American men. African American men suffer disproportionately from the disease, having a 50% higher incidence and a 2-fold greater mortality than do Caucasian men [8]. The reason behind this disparity is still unclear. Researchers must determine if it is socioeconomic issues, access issues, or biological issues that are creating this disparity. Unfortunately, it is unlikely we will learn much about screening in individuals of African descent from the European screening study, due to a low accrual of individuals of African descent. Though the numbers may be higher in the US screening study, we will still be left analyzing a subset of the cohort and thus dealing with data that may not be statistically robust. Until the true culprit of this disparity is identified, continued education and screening in hopes of early detection of prostate cancer in African American communities should continue.

Ultimately, however, I think the debate over prostate cancer screening is moot, since we have progressed beyond advocating treatment for all prostate cancers. With the aid of Partin Tables [9], Kattan Nomograms [10], assessing PSA velocity[11] or percent of prostate cores positive for prostate cancer [12], we continue to improve our ability to diagnose low-grade, low-stage, perhaps non-lethal prostate cancer that can be managed with expectant management. Numerous studies have demonstrated the feasibility of expectant management [13-15]. Expectant management is a treatment option that is currently under-utilized. Currently, there is an ongoing North American trial not only assessing the utility of expectant management, but also the ideal follow-up schema to ensure adequate monitoring of disease (START, Surveillance Therapy Against Radical Treatment, Laurence Klotz, principal investigator). Thus I recommend vigorous recruitment to this much needed trial that will provide treating physicians and patients much needed information on expectant management.

We are in the midst of the Information Age, where individuals realize that information or knowledge is power. In fact in order to gain a competitive advantage, various industry sectors are combing through reams and reams of secondary data prior to making critical decisions. The healthcare sector, specifically our patients, are no different. Today patients already come to their physicians' office after having done extensive research on the internet. These patients crave information regarding their specific condition. By having more information, patients are starting to realize that they can make a more informed decision about their care and this is what was always at the root of the prostate cancer screening dilemma.

Thus the true question is not whether we are going to screen for prostate cancer, we have progressed past this hurdle. The question of the day is when should we treat prostate cancer. Unlike for prostate cancer screening, in expectant management we do not even have significant circumstantial evidence to support this concept. Thus we anxiously await the results of the START trial assessing expectant management. So let us not continue to roll around in the quagmire of to screen or not to screen. Let's instead embrace the notion and the current trial assessing expectant management to answer the question, to treat or not to treat.

## Competing interests

The author declares that they have no competing interests.

## Pre-publication history

The pre-publication history for this paper can be accessed here:


